# Nonheritable Cellular Variability Accelerates the Evolutionary Processes of Cancer

**DOI:** 10.1371/journal.pbio.1001296

**Published:** 2012-04-03

**Authors:** Steven A. Frank, Marsha Rich Rosner

**Affiliations:** 1 Department of Ecology and Evolutionary Biology, University of California, Irvine, California, United States of America; 2 Department of Ecology and Evolution, University of Chicago, Chicago, Illinois, United States of America; 3 Ben May Department for Cancer Research, University of Chicago, Chicago, Illinois, United States of America

## Abstract

Recent cancer studies emphasize that genetic and heritable epigenetic changes drive the evolutionary rate of cancer progression and drug resistance. We discuss the ways in which nonheritable aspects of cellular variability may significantly increase evolutionary rate. Nonheritable variability arises by stochastic fluctuations in cells and by physiological responses of cells to the environment. New approaches to drug design may be required to control nonheritable variability and the evolution of resistance to chemotherapy.

## Abstract 2

Heritable genetic or epigenetic changes in cells are thought to drive tumor development, metastasis, and drug resistance. This essay discusses the possibility that nonheritable phenotypic variability contributes to the evolution of cancer, suggesting new approaches to treatment.

Cancer progression is a series of evolutionary changes. Those changes include enhanced cellular proliferation, reduced cellular death by abrogating normal apoptotic mechanisms, greater invasiveness by increased expression of proteases, and improved colonizing ability to achieve metastasis [1]. In response to drug treatment, cancer cells often evolve resistance and continue to spread.

Each evolutionary step typically proceeds by acquisition of genetic or heritable epigenetic changes in cells. When does the novel genetic change arise in an evolutionary step? By the current view, rare genetic variants arise before widespread phenotypic change. The idea is that a novel phenotype always comes from a novel genotype [2],[3].

For example, rare resistant genetic variants may exist before drug treatment [4]. The drug selects those rare resistant variants, killing the other cancer cells. In progression, a genetic mutation may abrogate apoptosis, allowing that genetic clone to expand. Genotype leads to phenotype leads to evolutionary change.

But does genotype always come before phenotype in an evolutionary response [5]? Consider the alternative in which phenotype comes first, before any genetic or heritable epigenetic change. In initial drug treatment, cancer cells with the same genotype may vary phenotypically with regard to resistance. Nongenetic phenotypic variation arises by stochastic fluctuations in cellular state or by cells responding physiologically to the changed environment.

Some of the phenotypic variants may be resistant, although not genetically or heritably different from the susceptible cells. In the absence of further treatment, the surviving cells would eventually produce the same range of phenotypes as before treatment. No evolutionary change has occurred. With repeated treatment, the novel selective pressure of the drug treatment may eventually select a new genetic variant among those initially surviving cells. At that point, evolutionary change occurs. Nongenetic phenotypic variability eventually leads to acquisition of a genetic variant and evolutionary change.

Phenotypic variability may also come before genetic variability during progression and metastasis. For example, in metastatic colonization, a subset of phenotypically variable cells among a population of genetically similar cells may survive initially. Among those survivors, the novel selective pressure of the new environment may eventually favor a new genetic variant, leading to evolutionary change.

It is certainly possible that nonheritable phenotypic variants come before genetic variants in cancer evolution. But does it actually happen that way? And if so, does it matter whether phenotypic or genetic variants come first in evolutionary progression and drug resistance? How does the particular ordering influence one's understanding of progression and the approaches one might use in treatment?

## Overview

In the past few years, many studies have directly measured the nonheritable phenotypic variability in populations of cells [6],[7]. Several articles have argued that nonheritable cellular variability may significantly influence the evolution of drug resistance or other key steps in cancer progression [8]–[11]. However, mainstream cancer research continues to emphasize the primary role of genetic variants or heritable epigenetic variants in initiating the evolutionary changes of cancer progression and drug resistance. The current literature on cellular variability, although interesting, has yet to make a convincing case for the fundamental role of nonheritable cellular variation in cancer.

We review some of the recent observations on cellular variability. We then extend that prior work in two ways.

First, we use fundamental concepts of evolutionary theory to show how nonheritable cellular variability likely plays a key role in the evolutionary steps of cancer progression and drug resistance. Nonheritable variability accelerates evolutionary rate particularly strongly when populations experience intense competition or face novel and extreme challenges for adaptation [5]. That intensity of competition and extremeness of environmental challenge characterize the evolutionary steps in cancer progression and drug resistance.

Second, we predict that cancer cells will often evolve to express greater nonheritable variability, because the evolutionary changes of carcinogenesis, metastasis, and drug resistance are more likely to occur in cellular populations that express enhanced variability.

## Cellular Variability

Nonheritable variability takes two forms. Stochastic cellular variability arises from random fluctuations in the numbers or functions of proteins. Phenotypic plasticity arises from the response of cells to the environment. Either form of variability may allow a cell to express a novel phenotype without genetic or heritable epigenetic change. The initial expression of phenotypic novelty accelerates subsequent heritable evolutionary changes. In this section, we briefly describe examples of cellular variability. In later sections, we turn to more detailed discussion of the evolutionary consequences.

Sigal et al. [12] measured stochastic variability in protein levels in human cells. They followed the dynamics of 20 proteins in individual cells over several cellular generations. They corrected for variations between cells in the stage of the cell cycle. After correction, the protein levels varied between cells, with most standard deviations between 15% to 30% of mean levels. High protein levels in a particular cell tended to decay over a few cellular generations. This reversion to the mean shows that cellular variability does not arise from intrinsic differences between cells. Instead, random fluctuations in protein levels between cells create heterogeneity in the population.

Many earlier studies of bacteria demonstrated stochastic variation in protein levels and phenotypes [7]. Those earlier studies, combined with the study by Sigal et al. [12] and other experiments [6],[13]–[16], demonstrate the significant levels of protein and phenotypic variability that arise from random fluctuations of cellular state. Those random fluctuations generate nonheritable cellular variability.

Alternatively, cells with a common heritable genome can generate different phenotypes by their response to the environment [17]. Changes in external stresses and in signals from other cells greatly alter cellular physiology and sometimes push cells to different developmental states [18],[19]. In a changed developmental state, a cell may take on the characteristics of different tissues or of a stem cell like proliferative capacity. In those altered states, cells often change in their ability to tolerate stress and to respond to signals for proliferation or apoptosis. These types of cellular plasticity potentially generate diverse and sometimes novel phenotypes without underlying genetic or heritable epigenetic change. The new nonheritable phenotypes can initiate evolutionary change [20], including resistance to drugs.

## Resistance of Cancer Cells to Chemotherapy

In the common theories of chemotherapy, resistance arises from rare mutant cells present before the start of treatment [4],[21]. Such preexisting genetic variation for resistance is expected in large cellular populations when single or double site mutations confer resistance.

With a combination of drugs applied simultaneously, the probability is very low that any cell contains all of the genetic variants necessary to protect against all of the treatments. As in current HIV treatment strategies [22],[23], combination chemotherapy minimizes the evolution of resistance particularly well when multiple mutations are needed for the initial expression of resistance. However, three recent studies of cancer find that, in particular cases, the origin of resistance begins with nonheritable cellular variation instead of preexisting genetic variants (Box 1). Each study emphasized a different mechanism that could generate nonheritable cellular variability: protein fluctuation, cellular signaling state, or histone-mediated alterations in cellular proliferation. Although these studies provide an intriguing suggestion of broader issues in the evolution of drug resistance, the role of nonheritable variability has yet to be integrated into widely understood conceptual or practical approaches to drug treatment design [8]–[10].

**Box 1. Drug Resistance Initiated by Nonheritable Cellular Variability**

Spencer et al. [14] showed that stochastic variability in protein levels and protein states explained the observed cellular variability in survival when exposed to the drug TRAIL (tumor necrosis factor-related apoptosis-inducing ligand), which can induce apoptosis in sensitive cells. Protein state is transmitted from mother to daughter cells, causing transient heritability. However, new protein synthesis causes a decay in heritability over a few cellular generations. Thus, the protein fluctuation can initiate resistance in the absence of stably heritable variants, but cannot by itself lead to widespread resistance. Subsequent genetic or stable epigenetic change is needed after the initial resistance expressed by stochastic cellular variability.

Although protein fluctuations correlate with cellular phenotype, the mechanisms of phenotypic variability may be more closely associated with fluctuations in states determined by signaling pathways. Singh et al. [35] measured heterogeneity in signaling states by colocalization patterns of activated signaling molecules from microscopy images. In lung cancer cells, differences in cellular signaling state correlate with the most sensitive and resistant populations in response to the drug paclitaxel. This study established that a significant fraction of the variability in signaling state was expressed by cells with a common genotype, suggesting that stochastic fluctuations in signaling state may cause the phenotypic heterogeneity in resistance.

Sharma et al. [36] observed small subpopulations of cells that survived treatment by a variety of anticancer kinase inhibitor drugs. The resistant cells had an altered chromatin state that depends on a particular histone demethylase. Drug tolerant variants typically constituted about 0.3%–0.5% of the initial population. Those cells that survive drug treatment are mostly quiescent. When those quiescent cells are grown in the absence of drug, they resume growth and rapidly regain drug sensitivity, demonstrating that resistance is a transient nonheritable phenotype. If resistant, quiescent cells are exposed to continued drug treatment, about 20% eventually resumed normal proliferation. Those proliferating cells required about 90 doublings in drug-free passage to restore drug sensitivity, suggesting that the nonheritable resistant state had become stabilized under continued drug treatment.

These studies are important, because initial drug treatments may be driving tumors to genetically based stable resistance by first selecting nonheritable phenotypes generated by cellular or tissue variability. If so, we may need to rethink approaches to treatment design. Before turning to aspects of treatment, we first explain how nonheritable variability accelerates the evolution of heritably based resistance.

## Nonheritable Variability and Evolutionary Theory

To move forward, we need a clear conceptual framework. How should the recent results on nonheritable variability in drug resistance be interpreted in relation to genetic variability? What is the broader significance in the context of other evolutionary steps in carcinogenesis? How should new experiments be designed in light of the potential role of nonheritable variability?

These questions are timely, because new technologies provide the tools to measure both genetic variability and nonheritable aspects of cellular variability. Those refined tools offer great opportunity, but we need clear principles to exploit that technology and to gain a deeper understanding of carcinogenesis and drug resistance. Because the problems concern evolutionary change of cells and tissues, the principles arise from evolutionary theory.

The idea that nonheritable phenotypic variability accelerates evolutionary rate has a long history. In cancer research, Rubin [20] clearly described observations and concepts in which nonheritable phenotypic variability initiates key steps in carcinogenesis. Rubin did not connect his ideas to classical evolutionary theory, instead roughly sketching out the logic as a novel view of cancer evolution. Later authors have repeated this argument for drug resistance or, more generally, for the evolutionary steps in the development of cancer [8]–[11]. Those later articles sometimes mention the literature from evolutionary theory, but do not develop that connection in a way that leads to specific predictions or novel insights.

Within the evolutionary literature, many recent reviews discuss the relation between nonheritable variation and evolutionary rate. The extensive history goes back to Baldwin [24] and includes several subsequent theoretical refinements [5],[25]–[33]. For our purposes, we can start by thinking of evolutionary adaptation as analogous to the problem of searching for an improved outcome in a complex space of alternatives [34]. With regard to cancer, “improved outcome” means modified cells or tissues that grow beyond normal restraints and may be regarded as outcompeting normal cells and tissues.

The key issue concerns how new traits arise [5]. How does the evolving system of cancer tissues find novel phenotypes that improve competitive success and cause the spread of the tumor? The search process for evolutionary novelty can be divided into short and long components that differ by the degree of phenotypic change required for cells and tissues to respond to environmental challenges [32].

“Short-range search” occurs when a cellular clone expresses a variety of phenotypes that differ by relatively small changes, such as altering ligand binding or modulating the pace of cell cycle progression. “Long-range search” occurs when cells derived from a clone express phenotypes that differ by relatively large changes, such as altered developmental state, tissue type, or other variant expression unlikely to occur as a stochastic phenotypic fluctuation. Short-range and long-range search play the role of discovery in the evolutionary search for novel traits. In addition to those two types of discovery process, “local adaptation” by genetic variation and selection refines the match between existing traits and the environment.

The following sections discuss each of these three components of evolutionary variability and adaptation. Box 2 shows how each component arises in the adaptive vertebrate immune system. Vertebrate immunity provides the best-known biological example of how the three aspects of variability and adaptation combine to determine the overall nature of evolutionary change. By studying vertebrate immunity, one may gain insight into the different processes of variability and adaptation in cancer evolution.

**Box 2. Synergism between Adaptive Processes in Vertebrate Immunity**

The mechanisms of antibody evolution in vertebrate immunity illustrate the synergism between long-range search, short-range search, and local adaptation [43],[44]. In the development of the immune system, B-cell lineages undergo programmed recombination early in life. That recombination yields numerous genetically distinct clones. Each clone produces an antibody with a distinctive pattern of binding affinities against different antigens. This initial generation of genetic diversity is a type of long-range search that creates novel and widely divergent phenotypes.

Each antibody type from this initial diversity tends to bind relatively weakly to a distinct set of antigens. The broader the range of binding affinities for a particular B-cell genotype, the more likely that the associated antibodies bind to a particular antigen. In this regard, the broad but relatively weak binding affinities of the original (natural) antibodies trade off the cost of weak binding for the benefit of a phenotypically diverse response. The diverse binding creates a form of short-range search spread over the binding propensity to a set of relatively similar antigens. This short-range search is a form of nonheritable phenotypic variability, because the different phenotypes arise from a common underlying genotype. Although the diversity of binding affinities for a B-cell clone is itself heritable, the binding of a particular B cell to a particular antigen does not heritably alter the range of phenotypes expressed by a daughter cell. Therefore, the range of binding affinities for a B-cell clone cannot by itself lead to evolutionary change within the B-cell lineage.

Upon challenge with a foreign antigen, those B cells with matching antibodies are stimulated to expand clonally. The B cells with initially weakly binding antibodies then undergo a programmed round of hypermutation to the antibody binding site and selection that favors those genetic variants that bind more tightly to the foreign antigen. This affinity maturation produces tightly binding and highly adapted antibodies in response to the novel challenge. Affinity maturation is the process of local adaptation.

The ability of the adaptive immune system to respond to the huge diversity of potential challenges depends on its synergism between genetic variability (long-range search) and the broad but weak binding of antibodies from each B-cell clone to a set of nearby antigens (short-range search). The initial natural antibodies arise from genetically diverse clones produced by recombination. That genetic diversity by itself could not cover the huge space of possible challenges. It is the short-range nonheritable phenotypic variability around each genetic variant that allows broad coverage against novel challenge. Once partial recognition is achieved by the natural antibodies, the system can refine the match locally by affinity maturation, which is a process of local adaptation by heritable variation and selection.

### Short-Range Search by Nonheritable Stochastic Variability

Suppose, for example, that cells with an altered signaling state can survive drug treatment [35],[36]. How does the altered signaling state first arise? If a clone of cells expresses a range of cellular signaling states, then some cells may, by chance, express a phenotype that can survive the initial challenge. Those surviving cells would not differ genetically from the killed cells. Instead, each surviving cell would produce daughter cells with roughly the same distribution of phenotypes as the initial cellular population. But those surviving cells could subsequently acquire genetic or heritable epigenetic variants that tuned the signaling process to the challenge of drug resistance.

The initial survival is by stochastic variability of phenotype. That variability is typically a short-range search, because the phenotypes sampled must be produced by a clone in which different cells express fluctuations around the underlying heritable type. Such fluctuations typically explore relatively nearby phenotypes, rather than creating dramatically new phenotypes.

Occasionally, a widely divergent phenotype may arise by fluctuations in expression. But a widely divergent phenotype is likely to be rare, and a rare survivor is unlikely to generate a population that could accumulate the stabilizing genetic or epigenetic changes needed for long-term survival and adaptation. Thus, nonheritable stochastic variability is most efficient at promoting adaptation with respect to favored phenotypes that are not too distant from the current type [32].

### Long-Range Search by Genetic Variability or Physiological Plasticity

Certain environmental challenges require novel phenotypes that are unlikely to arise by stochastic fluctuations and short-range search. For example, metastatic spread or new mechanisms of drug resistance may often demand a novel phenotype. The generation of long-range novelty must be rare. However, such novelty appears to occur in certain stages of carcinogenesis and perhaps in certain cases of resistance to drugs. How do such long-range shifts in phenotype arise and spread in populations in response to novel and sometimes extreme environmental challenge?

Genetic or stably heritable epigenetic variants can cause major shifts in phenotype. A large cellular population inevitably harbors many rare genetic variants. A novel challenge may favor one of those rare variants, allowing rapid adaptation to arise from a preexisting pool of heritable variants.

Alternatively, an altered environment may induce expression of a significantly altered phenotype [5],[17]. Such physiological plasticity in response to the environment can cause major shifts in phenotype. For example, a changed environment could drive cellular expression to a state that is common for a different type of tissue or a different stage in development. Or a novel environment could induce a phenotype not commonly expressed in normal tissues, including extreme stress responses or aberrant phenotypes arising from novel environmental conditions.

Long-range changes in phenotype by preexisting heritable variants or by environmental induction may allow cells to survive extreme or novel challenges. Those cells that survive the initial challenge may then adapt by subsequent nonheritable short-range search and by acquiring heritable genetic and epigenetic changes that enable local adaptation.

### Local Adaptation

Short- and long-range search discover novel phenotypes. In addition to discovery of novelty, adaptation also depends on small alterations to fine tune an existing phenotype and improve success. For example, if an aberrant tissue is already competing with neighboring tissue for limiting nutrients, an increase in the rate of nutrient uptake may often be favored by heritable variants that tune tissue physiology. Similarly, if an aberrant tissue is already secreting signals to attract complementary stromal cells, heritable variants may improve the efficiency of signal expression to attract stroma and enhance the competitive success of the tissue. Or a receptor may change slightly, preventing a drug from binding to and entering the cell. These sorts of tunings are a form of local adaptation. The changes do not produce novel characteristics, but instead improve the competitive match of the cell or tissue to its environment.

## Synergism between Different Adaptive Processes

Understanding cancer progression and drug resistance requires understanding the evolutionary processes that change cellular populations and tissues. We have emphasized three types of evolutionary process that differ by the underlying cause of phenotypic variability and by the amount of phenotypic change required for cells and tissues to respond to environmental challenges. These three processes are not mutually exclusive. In fact, the changes required to achieve drug resistance or the steps in carcinogenesis may often arise by synergism between the different adaptive processes.

Consider the problem of drug resistance. In several studies, empirical evidence suggests that preexisting genetic variants provide the phenotypic variability selected by novel drug-specific environments [4],[21]. At the same time, we described three experiments in which nonheritable phenotypic fluctuations provide the phenotypic variability that allows initial survival (Box 1). Many bacterial studies also show that initial survival in the presence of drugs depends on nonheritable phenotypic variability [37]–[39].

How can we reconcile the evidence for preexisting genetic variation with the observations on nonheritable phenotypic variability? Part of the answer is that, to some extent, one finds what one is looking for. Experimental designs that seek preexisting genetic variability will often find it, and experimental designs that seek nonheritable phenotypic variability will often find it.

Focusing on preexisting genetic variability versus nonheritable phenotypic variation may be a misleading contrast [5]. Instead, we think that a natural synergism occurs between the different types of variability [32]. Nonheritable physiological plasticity often provides a type of long-range search, in which extreme or novel environments induce novel phenotypes. Preexisting genetic variants can also generate novel phenotypes in long-range search when the genetic changes induce major phenotypic shifts. By contrast, nonheritable stochastic fluctuations often provide a type of short-range search. The complex process of evolving a phenotype that can survive an extreme or novel challenge may sometimes require both a long-range component to create a phenotype somewhere near a viable solution and a short-range component to provide additional phenotypic variability that closely matches the particular environmental challenge (Box 2).

Our main point is that genetic and nonheritable phenotypic variation are not alternatives. Rather, to understand the complex evolutionary processes in drug resistance and in the stages of carcinogenesis, one needs to consider the synergisms between different aspects of evolutionary search and adaptation.

## Open Problems

We described three experiments in which nonheritable phenotypic variability played a key role in drug resistance (Box 1). Those experiments, along with the theory we have developed, demonstrate that nonheritable variability can be important. But how important and widespread are the consequences of nonheritable variability? Study of the following open problems may help to answer that question.

### Strategies of Drug Treatment

Current research on drug treatment of cancer follows an analogy with successful HIV combination therapy [22],[23]. In an individual infected with HIV, the large viral population typically contains preexisting rare genetic variants resistant to any single drug. Each additional simultaneously applied drug reduces the chance that a single virus has all the necessary preexisting resistance variants or can generate the necessary mutations in the short time before clearance by the drug. Optimal treatment design comes down to choosing the right number and combination of simultaneously applied drugs to minimize both resistance and toxic side effects.

In cancer treatment, combination therapy has obvious benefits [40]. It will almost always be more difficult for a tumor to overcome additional simultaneously applied drugs. But the laboratory observations on drug resistance through nonheritable phenotypic variability raise an important question. Should combination therapies be designed differently if the origin of resistance sometimes comes from nonheritable variability rather than preexisting genetic variants? The short answer is that no one knows, partly because the question is rarely asked.

Consider first why cancer may differ from HIV. The HIV genome codes for nine transcripts and fewer than 20 proteins. The relation between genotype and phenotype is relatively simple. An amino acid substitution may, for example, alter the binding dynamics of the drug to replication enzymes or to proteases that process HIV gene products [41]. Human cells and tissues are far more complex phenotypically. The same human genome leads to all tissue types and to a wide range of physiological responses. The potential range of nonheritable phenotypic variability for human cells and tissues is vastly greater than for an HIV genotype.

Given the phenotypic range expressed by a human cell through stochastic fluctuations and in response to different environments, how might one think about alternative treatment strategies? The cancer cells that survive initial treatment may be those that are particularly good at responding to stressful environments by upregulating any one of a number of cellular stress responses, including cellular quiescence. If so, then the best two-drug treatment might be the combination of a drug that stresses and often kills cells and a stress response inhibitor that reduces the chance that some cells may survive the induced stress. In this case, the first drug targets a gene in a key pathway. The second drug targets a pathway that generates the nonheritable phenotypic variability that may allow initial survival of cells in response to stress caused by the first drug.

### Homeostasis and Variability

Many evolutionary steps in carcinogenesis and drug resistance are evolutionary responses to novel or extreme environments. Metastasis requires survival and growth in a new environment. Certain stages in carcinogenesis may require tolerance to hypoxia or acidosis. Whenever novel challenges arise that require altered phenotypes to survive, those cells and tissues that express a broader range of phenotypes will often be favored.

Drugs, metastatic spread, and other novel challenges select expression of increased nonheritable variability, because those cellular populations expressing a broader range of phenotypes have a greater chance of initial survival and subsequent adaptation. Thus, over the course of cancer progression and response to drugs, tumor cells may often evolve to express greater nonheritable phenotypic variability.

The range of nonheritable variability is itself a heritable trait. For example, increased expression of variability may arise from reducing or knocking out normal, heritable homeostatic mechanisms [42]. Reduced homeostasis is likely to cause greater stochastic perturbations in the expression of phenotype. Stochastic perturbations enhance the rate of adaptation when favored phenotypes are nearby, in the sense discussed above in terms of short-range search. These issues may lead to some interesting experimental approaches (Box 3).

**Box 3. Nonheritable Variation in Drug Resistance: Experimental Approaches**

Suppose, initially, that no cells have heritable resistance to a particular drug. To achieve heritable resistance, a cell must acquire a genetic change or a stable epigenetic change. One possibility is a single mutation that prevents the drug from binding to its target. Resistance by a single mutation is adaptation by a single jump. By contrast, if resistance requires multiple simultaneous changes, adaptation by a jump from the susceptible to the resistant state becomes harder. In both cases, the difficulty is that adaptation by a jump to a new phenotype may often be a rare event.

Now consider how the problem changes if we include nonheritable phenotypic variation. We use a numerical scale to give a sense of the issues, although resistance phenotypes may not be aligned along a single quantitative dimension. Suppose the initial susceptible genotype has an average phenotype of zero and a standard deviation of σ, varying according to a normal distribution. To achieve resistance, a cell must have a phenotype greater than three. If there is no phenotypic variation, σ = 0, then no cells of the susceptible genotype are resistant. Resistance can only be achieved by a mutation that causes a phenotypic jump above three. If σ>0, then a fraction of the susceptible cells achieves resistance by having a phenotype above three. For example, if σ = 1, then 0.1% of cells are resistant, if the standard deviation increases to σ = 2, then 6.7% of cells are resistant, and if σ = 3, then 15.9% are resistant.

A rise in the variance increases the number of surviving cells. However, the more interesting issue is that a rise in the variance changes the nature of the problem with respect to evolving a heritable increase in resistance. With no phenotypic variance, σ = 0, heritable resistance requires a mutational jump to a phenotype greater than three. By contrast, if σ = 1, then a mutation that increases mean phenotype from 0 to 0.1 raises the fraction of surviving cells from 0.135% to 0.187%; if σ = 2, then a mutation that increases mean phenotype from 0 to 0.1 raises the fraction of surviving cells from 6.7% to 7.4%. Phenotypic variance changes the nature of the adaptive problem. When there is no variance, a big heritable jump is needed. When there is variance, small heritable changes in phenotype can be favored, allowing the population of cells to adapt gradually to the drug challenge. Adaptation by small changes is generally easy and rapid, because small heritable variations are common. Adaptation by large jumps is often hard and unpredictable, because the process depends on whether a mutation causing a big jump can occur, and if so, how long one must wait for that mutation.

The theory is simple and, in the abstract, always true. But how the evolutionary process plays out in actual cellular populations can be difficult to predict. The hard part is to figure out what the proper phenotypic dimension is for understanding the adaptive problem. The experimental system with yeast developed by Levy and Siegal [42] provides a starting point. They created many single-gene deletion strains and used high throughput techniques to measure morphological variation for each deletion strain. They found that morphological variation increased in more than 300 of the single-gene deletion strains relative to a base “wild-type” strain.

A similar approach might be used to analyze the fraction of cells resistant to a drug for various single-gene deletions. The strains that have a relatively higher fraction of surviving cells may have increased phenotypic variance in the dimension that influences survival to the challenge. One could then analyze in more detail the evolutionary response of those strains with higher phenotypic variance, to determine if a resistance problem that initially required a large mutational jump had been transformed into an adaptive problem that responds by the accumulation of many mutations each of small effect.

That scenario and experiment focus on phenotypic variation created by stochastic fluctuations around a mean value. We have also emphasized that physiological responsiveness to novel environments can have a similar and often more powerful effect on the dynamics of adaptation. Greater physiological responsiveness tends to speed adaptation by reducing the size of the heritable phenotypic jumps needed to increase fitness. This problem is challenging experimentally, because one needs to find a system in which physiological responsiveness itself varies with respect to the dimension of the environmental challenge imposed by a selective process, such as a drug treatment. Perhaps a high throughput approach could be developed that is similar in concept to that of Levy and Siegal. One could initially screen for the degree of responsiveness of cells to environmental challenge. Then, one could follow with studies on the rate of evolution of drug resistance, comparing the evolutionary rate between cells that have greater or less physiological responsiveness. That kind of study might identify the characteristics of physiological responsiveness that are most important for the evolution of drug resistance, suggesting alternative targets for therapy.

Another way to increase variability is to respond more strongly to environmental change. For example, cellular populations that more easily change tissue type or developmental state in response to an altered environment also express greater variability. Such variability through enhanced response to environmental change increases the chance of success to novel challenge. Large changes in phenotype in response to novelty or stress enhance long-range search and can greatly enhance evolutionary rate.

The evolutionary challenges of carcinogenesis and drug resistance likely favor cellular populations with increased stochasticity by reduced homeostasis and increased capacity for environmental responsiveness. The associated nonheritable phenotypic variability increases evolutionary rate by enhanced short-range and long-range search.

Alternatively, certain challenges in carcinogenesis may favor reduced expression of nonheritable phenotypic variability. Suppose, for example, that rapid resource acquisition and cell division allow cells to outcompete neighbors. Expression of variability to explore alternative phenotypes would be a disadvantage, because those phenotypes best tuned to the local conditions win the race. Put another way, local adaptation to a fixed environment often favors a narrowing of phenotypic variability, which may alter subsequent evolutionary response to novel challenges.

### Synergism between Long-Range and Short-Range Search

There is a natural tendency to dichotomize the problem of cancer evolution into genetic mutations versus nonheritable variability. Do we need to think mainly about genetic variants, or does nonheritable variability initiate the key steps of carcinogenesis and resistance? However, both the theory and the analogy with vertebrate immunity (Box 2) suggest that a more nuanced approach may be needed to understand the evolutionary processes that drive carcinogenesis, metastasis, and drug resistance.

Suppose, for example, that the resistance to a particular drug requires preexisting mutations in the tumor population. That fact does not exclude the importance of nonheritable variability. As in vertebrate immunity, the genetic variant may bring the phenotype somewhere near what is needed to survive. But actual survival may require a physiological adjustment of the mutated cells to the stress induced by the drug. In this case, initial survival depends on the synergism between the mutation and an upregulated stress response. Alternatively, there may be a synergism between the mutation and some other form of enhanced physiological responsiveness.

There is no direct evidence for such synergism, but few researchers are looking for it. Drug resistance is an evolutionary process, and evolutionary theory suggests that such synergism would greatly enhance the rate of adaptation to drug treatment. So the notion of synergism is at least a reasonable hypothesis that should be studied.

## Conclusions

The current cancer literature is dominated by the view that genetic or stably heritable epigenetic variants initiate the key evolutionary changes in carcinogenesis and drug resistance. Against that view, our theme is that nonheritable cellular variability often plays a key role in initiating the major evolutionary steps of carcinogenesis and drug resistance.

The main point is that tumorigenesis and drug treatment typically impose novel or extreme environmental challenges to cells and tissues. The rate of evolutionary change in response to such challenges is greatly enhanced by nonheritable phenotypic variability, often acting synergistically with genetic variability. A clear understanding of cancer and its treatment requires closer attention to these fundamental evolutionary processes.

Our view has two important implications for drug treatment design. First, the mechanisms of tumor resistance may combine genetic mutations and nonheritable phenotypic fluctuations. If so, then combinations of drugs should target both the likely genetic resistance mutations and the likely nonheritable resistance mechanisms, rather than solely targeting genetic mutations.

For example, a drug may target a particular cellular protein. Resistance may arise by a mutation in the targeted cellular protein or by nonspecific stress responses that compensate for the loss of function of the targeted protein. Drug combinations for both the targeted protein and the nonspecific stress response may be required. Alternatively, initial resistance to a drug that targets a particular protein may arise by nonheritable variability in rate of cellular proliferation. A drug combination that targeted the particular protein and also reduced fluctuations in cell cycle state may be effective.

The second implication for treatment arises when drugs drive cellular populations to a resistant state via a nonheritable intermediate state. For example, those cells initially resistant to a treatment may, by purely nonheritable phenotypic fluctuation, express a greater stress response than those cells that die. With continued treatment, some of those initially resistant cells may subsequently acquire a heritable change that upregulates the stress response. Those mutated cells will tend to increase, causing the heritable fixation of a generalized resistance mechanism. Subsequent alternative drugs may then perform poorly. In this case, initial drug combinations should include stress response inhibitors.

In these two examples, we have mentioned stress response as a form of nonheritable resistance. We chose that response because it is easy to understand how resistance to stress may enhance resistance to drugs. However, our point is more general. Any physiological mechanism that promotes nonheritable phenotypic variability in resistance tends to enhance evolutionary rate, including the rate at which stably heritable drug resistance emerges. Closer attention to the broad range of mechanisms that generate nonheritable phenotypic variation is likely to improve our understanding of resistance and the design of combination treatments.
